# Computational Studies of the Structural Basis of Human RPS19 Mutations Associated With Diamond-Blackfan Anemia

**DOI:** 10.3389/fgene.2021.650897

**Published:** 2021-05-24

**Authors:** Ke An, Jing-Bo Zhou, Yao Xiong, Wei Han, Tao Wang, Zhi-Qiang Ye, Yun-Dong Wu

**Affiliations:** ^1^State Key Laboratory of Chemical Oncogenomics, Peking University Shenzhen Graduate School, Shenzhen, China; ^2^Shenzhen Bay Laboratory, Shenzhen, China; ^3^College of Chemistry and Molecular Engineering, Peking University, Beijing, China

**Keywords:** Diamond-Blackfan Anemia, RPS19, missense mutation, structure stability, interaction, pathogenesis, ribosomopathy

## Abstract

Diamond-Blackfan Anemia (DBA) is an inherited rare disease characterized with severe pure red cell aplasia, and it is caused by the defective ribosome biogenesis stemming from the impairment of ribosomal proteins. Among all DBA-associated ribosomal proteins, RPS19 affects most patients and carries most DBA mutations. Revealing how these mutations lead to the impairment of RPS19 is highly demanded for understanding the pathogenesis of DBA, but a systematic study is currently lacking. In this work, based on the complex structure of human ribosome, we comprehensively studied the structural basis of DBA mutations of RPS19 by using computational methods. Main structure elements and five conserved surface patches involved in RPS19-18S rRNA interaction were identified. We further revealed that DBA mutations would destabilize RPS19 through disrupting the hydrophobic core or breaking the helix, or perturb the RPS19-18S rRNA interaction through destroying hydrogen bonds, introducing steric hindrance effect, or altering surface electrostatic property at the interface. Moreover, we trained a machine-learning model to predict the pathogenicity of all possible RPS19 mutations. Our work has laid a foundation for revealing the pathogenesis of DBA from the structural perspective.

## Introduction

Diamond-Blackfan Anemia (DBA, OMIM # 105650) is an inherited rare pure red blood cell aplasia (∼5 to 7 per million birth) (Vlachos et al., 2001; Da Costa et al., 2018) characterized by the failure of erythropoiesis but with normal production of leukocytes and platelets in the bone marrow (Diamond, 1938; Ball, 2011; Da Costa et al., 2018; Engidaye et al., 2019). It usually presents during the first year of life, and affects the follow-up growth and development, resulting in short stature and congenital abnormalities. An elevated risk of cancer with ∼4.8-fold is observed as well (Vlachos et al., 2018). Currently, steroids and blood transfusions can keep the disease at bay but with considerable side effects, and the only cure for the bone marrow failure phenotype of DBA is hematopoietic stem cell transplantation, but donors are often unavailable (Engidaye et al., 2019).

In DBA, almost all linked genetic lesions come from genes encoding ribosomal proteins (RP) (Aspesi and Ellis, 2019; Farley-Barnes et al., 2019), whose haploinsufficiency is believed to impair ribosome biogenesis. Ribosomes are the protein translation machines, and are intensively implicated in the processes with high requirement of protein synthesis, such as hematopoiesis and embryonic development (Cmejla et al., 2000). During the process of promoting erythroid lineage commitment from hematopoietic stem and progenitor cells, the quantity of ribosomes plays a key role (Khajuria et al., 2018). The impaired ribosome biogenesis will lead to decreased ribosome quantity and thus the failure of erythropoiesis (Engidaye et al., 2019; Ulirsch et al., 2019).

DBA is mainly inherited in an autosomal dominant manner and caused by loss-of-function mutations (Engidaye et al., 2019). All DBA-related RP gene mutations identified to date are heterozygous (Da Costa et al., 2018), indicating that homozygous RP gene mutations are lethal. The homozygous lethality of RP genes has been supported in several animal models including zebrafishes and mice (Amsterdam et al., 2004; Matsson et al., 2004).

Among all the RP-coding genes, *RPS19* have affected the most majority of DBA patients (∼25%) (Ulirsch et al., 2019). As an indispensable component of the ribosome small subunit (SSU or 40S subunit), RPS19 interacts with the 18S rRNA and other RPs in the mature SSU, contributing to the assembling and stability maintenance of SSU (Gregory et al., 2007; Ameismeier et al., 2018). During the biogenesis of SSU, RPS19 also plays essential roles in pre-rRNA processing, exportation of SSU precursors from nucleus to cytoplasm, and conformation maturation of 18S rRNA (Leger-Silvestre et al., 2005; Flygare et al., 2007; Juli et al., 2016; Duss et al., 2019).

With the continuous efforts that have been put in genetic screening and clinical studies of DBA, a considerable number of pathogenic RPS19 mutations have been cataloged (Boria et al., 2010; Khajuria et al., 2018), accounting for the most majority of DBA mutations (Ulirsch et al., 2019). In order to develop better diagnostics and treatment, it is swiftly becoming necessary to reveal the molecular basis of the pathogenic mutations (Kato et al., 2003; Alexov and Sternberg, 2013; Stefl et al., 2013). While it is usually straightforward to interpret how frameshift and splice mutations result in defected RPS19 and thereby its haploinsufficiency, it is more challenging to decipher missense pathogenic mutations of RPS19.

Over the past two decades, the consequences of a few missense mutations on the *RPS19* gene have been studied experimentally (Cmejla and Cmejlova, 2003; Da Costa et al., 2003; Gazda et al., 2004; Angelini et al., 2007). Gregory et al. (2007) solved the crystal structure of an archaea RPS19 (from *P. abyssi*), which shares 36% sequence identity with human RPS19, and then analyzed 16 missense DBA mutations based on it. These mutations were classified according to the impact on protein folding or on surface properties, and this binary classification has been applied to interpret pathogenic mutations in others’ work (Angelini et al., 2007; Campagnoli et al., 2008). However, the indirect conclusion deduced from the archaea RPS19 was not applicable to mutations occurring at the sites that are not conserved between human and *P. abyssi*, and the interaction details between RPS19 and other molecules were yet unavailable due to the lack of complex structures accordingly. Thanks to the recent development of Cryo-electron microscopy (Cryo-EM), several human ribosome structures have been determined and have thus created opportunities of further direct analyses (Anger et al., 2013; Behrmann et al., 2015; Khatter et al., 2015; Quade et al., 2015; Myasnikov et al., 2016; Zhang et al., 2016; Natchiar et al., 2017; Weisser et al., 2017; Ameismeier et al., 2018). A recent study briefly described that DBA missense mutations appear to predominantly disrupt the stability of RPS19 by altering the hydrophobic core or to perturb interactions with rRNA in assembled ribosomes (Ulirsch et al., 2019), but the specific approaches through which the mutations affect RPS19 remain unclear. Till now, an in-depth and systematic understanding of all available DBA missense mutations is still lacking, and a method for inferring the pathogenicity of newly identified RPS19 mutations is also highly required.

In this work, we conducted a comprehensive study on the structural basis of the DBA mutations from human RPS19 based on its 3D structures. First, starting with the human RPS19 structure extracted from the ribosome complex, we identified its main structure elements: the hydrophobic core with a bundle of five helices, two β-hairpins, and three putative intrinsic disordered regions (IDR). Second, we revealed that RPS19 interacts with 18S rRNA in the mature SSU through five conserved surface patches. Third, we identified several specific approaches through which DBA mutations destabilize the protein structure or affect interactions, and thereby lead to defected RPS19. Last, based on the understandings of the structural basis of DBA mutations, we trained a support vector machine (SVM) model to predict the pathogenicity of all possible missense mutations of RPS19, which will be valuable in future studies when new mutations are identified.

## Results

### Analyses of Human RPS19 Structure

An in-depth understanding of structure is the basis of studying mutations in a structural context, so we investigated the structure of human RPS19 in detail. Its structures both in free state and in the SSU complex were inspected in order to obtain an accurate description of its secondary structure, hydrophobic core, and interaction with other molecules.

#### Structure Elements of Human RPS19

**FIGURE 1:**
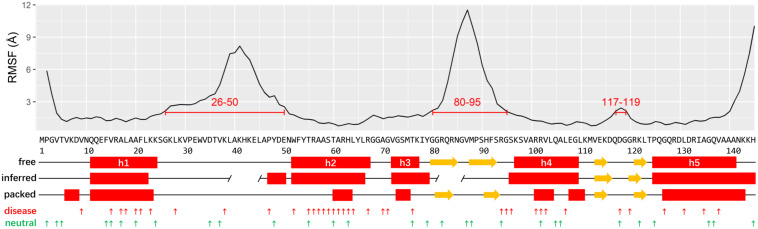
The plots of RMSF, structure elements, IDRs, and mutations of human RPS19. The putative IDRs (RMSF > 2 Å) are marked by red lines. The secondary structures are assigned based on the human RPS19 structure in free state, packed state, and inferred from *P. abyssi* RPS19, respectively (red rectangle: helix; yellow arrow: β-strands; gap in “inferred:” putative IDR inferred from *P. abyssi*). The DBA and neutral mutations are indicated with small upward arrows in red and green, respectively.

The recent determined Cryo-EM structure of human ribosome can be used as a starting point to assign the secondary structure of RPS19 (Ameismeier et al., 2018). Considering that RPS19 is packed in the ribosome complex enriched with negatively charged rRNAs, its conformation may be skewed from the free state. To obtain its conformation in free state, we have thereafter performed a molecular dynamics (MD) simulation of human RPS19 without the presence of 18S rRNA and other proteins. The follow-up conformation clustering resulted in seven clusters, where the largest cluster represents 70.1% of all conformations sampled in the whole simulation. The representative conformation of the largest cluster was adopted for the assignment of secondary structure. Our assignment confirms the existence of five helices (h1–h5) in free state but with boundary shifts of 3–4 residues compared with those inferred based on the archaea RPS19 structure previously (Gregory et al., 2007; Figure 1). Similar to the archaea RPS19, these five helices fold into a bundle and constitute the hydrophobic core (Supplementary Figure 1A). The comparison of RPS19 between in free state and packed in ribosome shows that the helix regions (defined according to the free state conformation) are more stretched (Supplementary Figure 2) and less regular in the packed state (Figure 1), indicating a higher winded level that may stem from the packing in the ribosome. Two β-hairpins can also be observed (Figure 1).

Two IDRs have been roughly proposed based on the archaea RPS19 structure previously (Gregory et al., 2007; Figure 1). Here we superposed the 7 representative conformations of the conformation clusters, and observed that two long regions (between h1 and h2, and between h3 and h4) display much higher flexibility than the helices (Supplementary Figure 1B). The RMSF plot also shows large fluctuations in these two regions (Figure 1). These observations are consistent with the existence of the two putative IDRs mentioned above, but they are much larger than previously inferred (Gregory et al., 2007). A third small IDR was also observed from the RMSF plot which was between h4 and h5. In accordance with our knowledge about the IDR’s preference for interaction, these IDRs bind 18S rRNA and other proteins in the assembled ribosome (see below). Notably, the IDR between h3 and h4 and the IDR between h4 and h5 overlap with the β-hairpins identified in the representative conformation, possibly suggesting that these β-hairpins may have much flexibility as a whole.

#### Analyses of Interactions in Packed State

Through analyzing the interaction interface of RPS19 in the complex of ribosome SSU, we found that RPS19 mainly interacts with 18S rRNA, RPS16, and RPS18 (Supplementary Table 1). The largest interaction interface is found between RPS19 and 18S rRNA, and it consists of 75 residues of RPS19 and 61 nucleotides of 18S rRNA, accounting for 51.7% of RPS19 and 3.7% of 18S rRNA, respectively (Supplementary Table 1). According to the definition of the secondary structure of human 18S rRNA (Petrov et al., 2014), five elements are involved in the interaction interface (Table 1). The interface is stabilized by 46 inter-molecule hydrogen bonds, which are enriched at the h41es10 and h41 of 18S rRNA. As for the interaction interfaces formed with RPS16 and RPS18, the areas are much smaller as shown in Supplementary Table 1.

**TABLE 1:** The detailed interactions between 18S rRNA and RPS19.

Interacting elements of 18S rRNA	Interacting residues of RPS19	Patch
h42 (1,603–1,607 and 1,626–1,629)	V37, K38, L39, K43, E44, L45	I
h41 (1,537–1,543 and 1,583–1,596)	P47, W52, T55, R56, S59, R62, H63, R67, Y79, G80	II
h42 and h43 (1,653–1,656 and 1,664–1,666)	R84, N85, G86, P89, H91, F92	III
h41es10 (1,561–1,571)	G71, V72, R94, S96, K97, S98, R101, R102, Q105, G120, R121	IV
h39es9 (1,414–1,430)	P2-V9, Y65, R129, D132, A135	V

**FIGURE 2:**
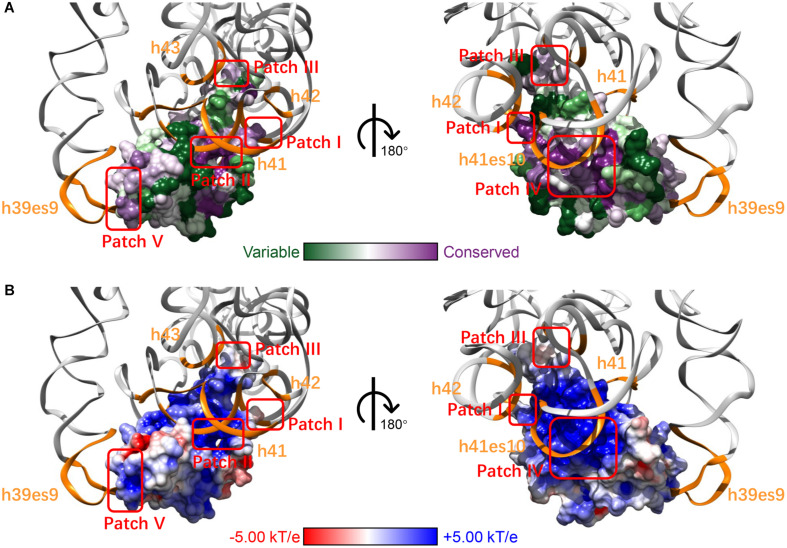
The five surface patches of RPS19 interact with the five secondary structural elements of 18S rRNA (orange) accordingly, with different color schemes rendering **(A)** conservation level and **(B)** electrostatic potential.

After mapping the conservation score to the solvent-excluded molecular surface by specific color scheme (Baker et al., 2001; Schrodinger, 2015; Ashkenazy et al., 2016), we further analyzed the interaction interface of RPS19 (Figure 2A). Five conserved patches (I, II, III, IV, and V) were identified by visual inspection, and they interact with the five secondary structural elements of 18S rRNA accordingly (Figure 2A and Table 1). Patch I and III correspond to the first two IDRs, respectively; Patch II is composed of the residues from the hydrophilic side of h2 and their nearby residues; Patch IV comprises the residues from the hydrophilic sides of h3 and h4, and the C-terminal of the third IDR; Patch V consists of the N-terminal coil of RPS19 and the exposed residues of h5. Moreover, all these 5 conserved patches present positive electrostatic potential (Figure 2B), which is well matched with the negatively charged rRNA at the interaction interface.

Previously, Gregory et al. (2007) had inferred that two highly conserved basic patches on the RPS19 surface might interact with rRNA. Patch I and III in our work have covered them, and three new patches were identified additionally. Moreover, our analyses have extracted the specific interaction residues and nucleotides between human RPS19 and 18S rRNA directly, thereby providing additional structural insights.

### Overview of DBA Mutations

**FIGURE 3:**
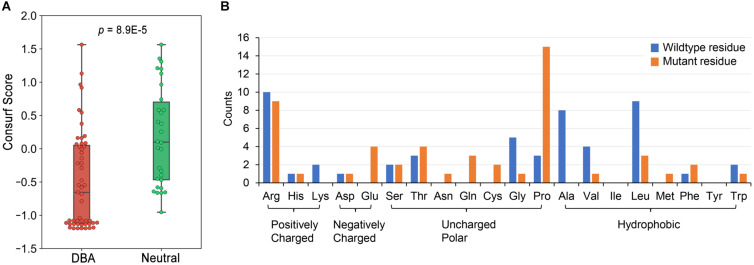
The conservation properties of RPS19 mutations and the residue type counts of DBA mutations. **(A)** The Consurf score boxplots between DBA and neutral mutations. The lower the Consurf score, the higher the conservation. **(B)** The count of wildtype and mutant residues in DBA mutations.

We have curated 51 DBA missense mutations (Supplementary Table 2) and 30 neutral ones (Supplementary Table 3). These data presumably constitute a very complete missense mutation dataset of RPS19 until now. We first checked the conservation features of DBA mutations. According to the calculated Consurf scores (Ashkenazy et al., 2016), the conservation levels of DBA mutation sites are significantly higher than the neutral ones (median: -0.665 vs. 0.0995, *p* = 8.9E-5, one-tailed Mann-Whitney U test, Figure 3A). Nearly one half of DBA mutations are located at the highly conserved sites (Consurf score < -1.0). These results are consistent with the general knowledge that disease mutations tend to occur at conservative sites (Kumar et al., 2009; Lal et al., 2020), which are indicative of functional or structural importance. Although it shows the potential in discriminating DBA and neutral mutations, the conservation feature cannot provide specific clues concerning the pathogenesis. We then analyzed the residue types and counts of the DBA mutations.

Overall, DBA mutations are more likely to occur at positively charged, hydrophobic or small residues (Figure 3B and Supplementary Table 2). Arginine (Arg) is the most mutated residue, accounting for about 19.6% (10/51) of all DBA mutations. Leucine (Leu) is the second most mutated residue (17.6%, 9/51), and small volume residues such as Glycine (Gly) and Alanine (Ala) are also mutated frequently (Figure 3B). We speculate that these Arg sites may participate in interactions with negatively charged rRNAs, and their mutations may disrupt or weaken these interactions. As for Leu, the speculation is that they often form the hydrophobic core, and mutations to polar residues may disturb the stability of the protein. Mutations from small residues to large ones may also influence the stability due to steric hindrance effect.

One striking feature is that a larger number of residues (29.4%, 15/51) have been mutated into Proline (Pro) (Figure 3B), a well-known helix breaker due to its cyclic side chain (a pyrrolidine ring) (Woolfson and Williams, 1990). Considering that the hydrophobic core of RPS19 is mainly composed of the bundle of the five helices, these mutations will presumably affect protein structure stability by destroying helices. There were also many other residues mutated to Arg (Figure 3B), most of which were mutated from hydrophobic residues, such as Leu and Tryptophan (Trp) (Supplementary Table 2). The hydrophobic effect of these residues would be disrupted by charged Arg, and thus protein folding would be affected.

Based on the analyses concerning the residue types, we can roughly infer that DBA mutations may mainly disrupt RPS19 from two perspectives: protein folding stability and interaction, as other studies have done (Zhang et al., 2010; Peng et al., 2018) or reviews have summarized (Stefl et al., 2013; Yates and Sternberg, 2013). However, more systematic data mining is required to consolidate these inferences, and more detailed mechanisms need to be explored in order to answer how a DBA missense mutation destabilize RPS19 or perturb its interactions. Compared to sequence, structure is more directly related to function. In the following analyses, more in-depth investigations in structural contexts have thus been conducted.

### Structural Basis of DBA Mutations

#### Substantial DBA Mutations Decrease Structure Stability by Two Approaches

**FIGURE 4:**
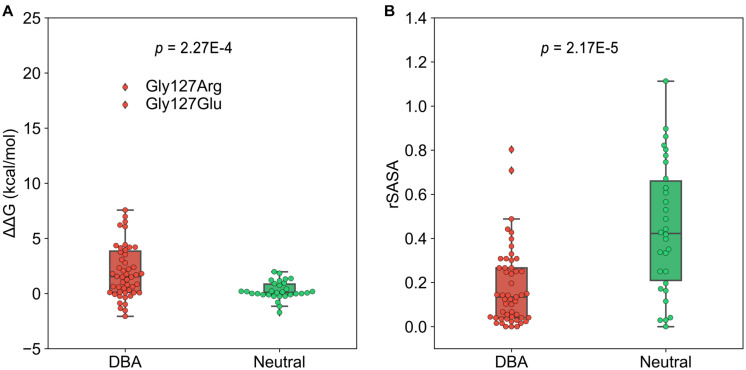
The boxplots of ΔΔG and rSASA. **(A)** The comparison of the ΔΔG caused by mutations. **(B)** The comparison of the rSASA of mutated sites.

To investigate to what extent the mutations affect stability of the protein structure, we calculated the change in free energy (ΔΔG) of folding of RPS19 caused by mutations using FoldX, a protein design algorithm that uses an empirical force field (Schymkowitz et al., 2005). We found that DBA mutations are more capable than neutral ones of decreasing the structural stability (median: 1.55 vs. 0.106, *p* = 2.27E-4, one-tailed Mann-Whitney U test, Figure 4A). There were 30 DBA mutations with ΔΔG greater than 1 kcal/mol, which was usually used as the cut-off to distinguish destabilizing mutations (Buss et al., 2018). This result has quantitatively demonstrated that the majority of DBA mutations should have reduced the stability of RPS19.

We further investigated the approaches by which DBA mutations decrease the structure stability. The first is the destruction of hydrophobic core. We compared the relative solvent accessible surface area (rSASA) between DBA and neutral mutations (Figure 4B), and found that DBA mutation sites are more buried than the neutral ones (median: 0.13 vs. 0.42, *p* = 2.17E-5, one-tailed Mann-Whitney U test). Further restricting in the 30 DBA mutations with ΔΔG > 1 kcal/mol shows even smaller rSASA values (median: 0.061). We checked the residue types of these 30 mutations, and found two major classes: 13 are mutated from typical hydrophobic residues (Leu, Trp, Val, and Phe) to other types with 22.96% hydrophobicity decreased on average (Rose et al., 1985), and 7 of them are mutated from small residues (Gly, Ala) to larger ones with 108.36% volume increased on average (Supplementary Table 2; Zamyatnin, 1972).

**FIGURE 5:**
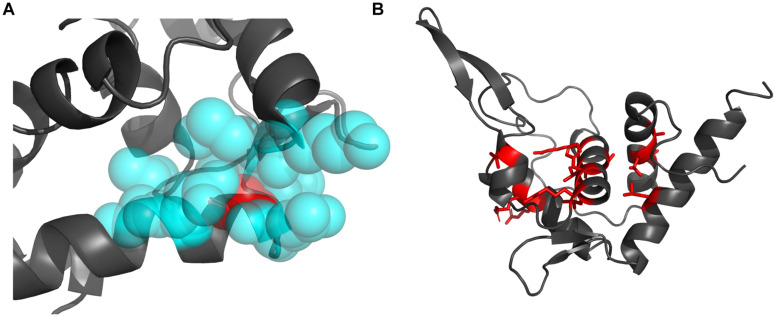
The structural visualization of the Gly127 and the sites mutated to Pro. **(A)** The locations of the Gly127 (red sphere) and its surrounding residues (blue sphere). **(B)** The locations of residues mutated to Pro (red sticks).

The decrease of hydrophobicity can weaken the hydrophobic interaction during protein folding, and the increasing of residue volume can result in steric hindrance effect. Either of them could destroy the hydrophobic core and thus lead to reduced stability (Liu et al., 2000; Loladze et al., 2002). The best examples are the two mutations occurring at Gly127 (Gly127Arg, Gly127Glu). Gly127 is located at the N-terminal of h5 and is fully buried (rSASA = 0, Figure 5A). Its surrounding residues (Lys111, Met112, Leu123, Thr124, Gln126, Asp130, and Leu131) forms a crowed interior space, which cannot accommodate larger residues. Therefore, the substitutions from Gly, which is the smallest residue (volume = 60.1 Å^3^), to larger Arg and Glu (volume = 173.4 and 138.4 Å^3^) would pose significant steric hindrance effect with 188.52 and 130.28% volume increase, respectively. Moreover, Arg and Glu have charged side chains with high hydrophilicity, which will also disturb the formation of the hydrophobic core during protein folding. It should be these two effects that cause Gly127Arg and Gly127Glu to result in the largest structural destabilization with ΔΔG = 18.72 and 17.12 kcal/mol, respectively (Figures 4A, 5A).

Secondly, as a helix-dominant structure, RPS19 can be destabilized by disturbing the folding of helices. Pro is well-known as a helix breaker (Levitt, 1978; Woolfson and Williams, 1990), which disrupts two adjacent hydrogen bonds and whose pyrrolidine ring pushes the preceding turn of backbone away by about 1 Å (Richardson and Richardson, 1988). Among the 30 DBA mutations with ΔΔG > 1 kcal/mol, eight are mutated to Pro. In the whole dataset, more than one quarter of disease mutations (29.4%, 15/51) are mutated to Pro (Supplementary Table 2), and there exists at least one such mutation in each helix (Figure 5B). These mutations may distort the folding of helices, disturb the formation of the helix bundle, and thus decrease the stability of RPS19. Many of these mutations (Leu64Pro, Ala20Pro, Thr76Pro, Arg102Pro, and Leu131Pro) were also proposed to affect the protein’s stability through breaking the folding of helices in other studies (Campagnoli et al., 2008; Ulirsch et al., 2019).

#### Numerous DBA Mutations Disrupt Interactions With 18S rRNA by Three Avenues

In section “Analyses of Interactions in Packed State,” we have identified 5 conserved surface patches that are involved in the interactions with 18S rRNA (Table 1). There are 24 of 51 DBA mutations are located in these patches, indicating that interfering with RPS19-18S rRNA interactions serves as another main feature of DBA mutations. When the RPS19-18S rRNA interactions are perturbed, the SSU assembling will be affected and thereby lead to insufficient biogenesis of ribosomes.

**FIGURE 6:**
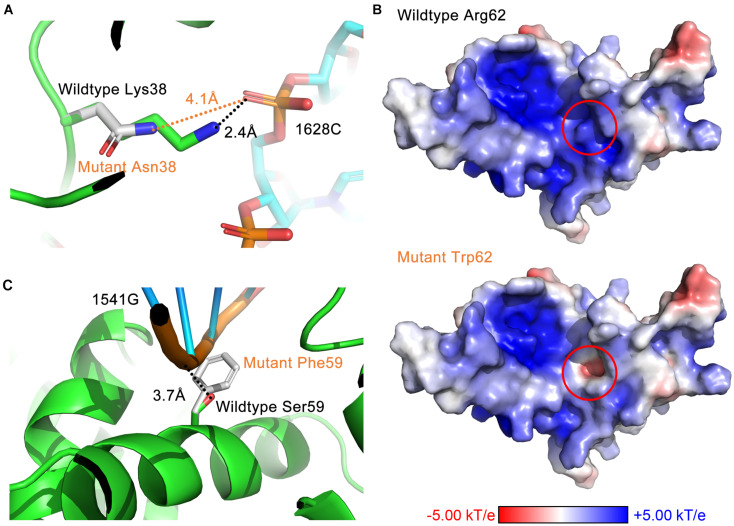
The three avenues of affecting interactions by DBA mutations. **(A)** Destroy hydrogen bonds by increasing the distance between bonding atoms (Lys38Asn). **(B)** Reverse the surface electrostatic properties (Arg62Trp). **(C)** Introduce steric hindrance at the binding interface (Ser59Phe).

Through analyzing the 3D structure, we have identified three avenues of affecting interactions. First, residue substitutions caused by DBA mutations may disrupt the hydrogen bonds formed between RPS19 and 18S rRNA, such as losing hydrogen donor or acceptor, increasing the distance between bonding atoms, and distorting the bond angle to unfavorable situations. The RPS19-18S rRNA interaction is stabilized by 46 hydrogen bonds (Supplementary Tables 1, 4), and about 16 DBA mutations break some of these hydrogen bonds (Supplementary Table 5). For example, mutation Lys38Asn in surface Patch I (Figure 6A) and Arg101Cys in surface Patch IV result in shorter side chains that lead to unsuitable distance between the potential bonding atoms; mutation Arg94Leu in Patch IV loses the hydrogen donor; and mutation Arg62Gln in Patch II distorts the bond angle to an unfavorable situation (Supplementary Table 5).

Second, DBA mutations may alter the surface electrostatic properties of the protein. Considering the prevalent electrostatic interactions between negatively charged rRNA and positively charged surface patches of RPS19 (Figure 2), we can speculate that DBA mutations in these patches may perturb them. For example, mutation Arg62Trp in Patch II have reversed the surface electrostatic potential from positive to negative, affecting the interaction between this site and h41 of 18S rRNA (Figure 6B). Although Trp itself is nearly electrostatic neutral, several nearby aromatic residues (Phe14, His63, and Tyr65) forming potential π–π stacking and several negatively charged or polar residues (Gln11, Gln12, and Glu13) may have contributed to the negative electrostatic potential at this site. Another example is the mutation Gly71Glu in Patch IV, which changed the surface electrostatic potential from positive to neutral, and thus perturbed the interaction between this site and h41es10 of 18S rRNA (Supplementary Table 5).

Last, a set of DBA mutations occurring at the interaction interface do not influence the interaction through hydrogen bonds or other electrostatic perturbations, but they substitute small residues into large ones, which will introduce steric hindrance at the binding interface. One of the examples is Ser59Phe in Patch II, which will perturb the interaction with h41 of 18S rRNA (Figure 6C). Another example in Patch II is the mutation Thr55Met, which will lead to the spatial collision between it and h41 of 18S rRNA. A previous experimental study showed that Thr55Met partially impairs the function of RPS19, but the mechanism was not clear yet (Aspesi et al., 2018). Here, our study has proposed a possible mechanism to explain this effect.

**FIGURE 7:**
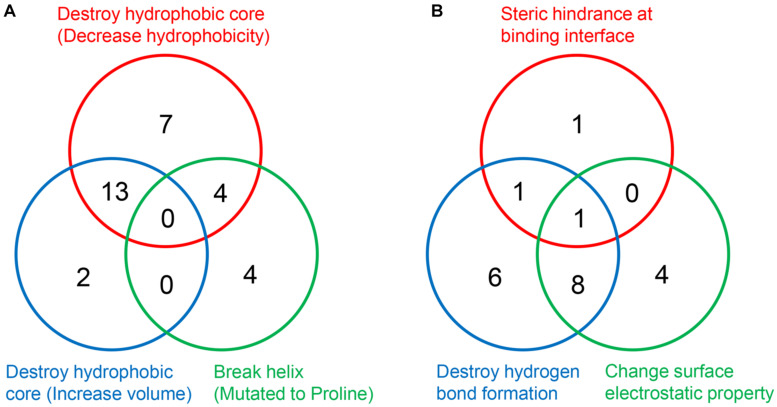
The number of DBA mutations with different structural basis. **(A)** Decrease structure stability. **(B)** Disrupt interaction with 18S rRNA.

It is worth noting that a mutation may disrupt the hydrophobic core or perturb the interaction through more than one approach (Figures 7A,B). Taken Ser59Phe as an example, it not only results in the steric hindrance effect, but also breaks a hydrogen bond. Specifically, when substituted to Phe from Ser, the residue volume has increased by 134%. On the other hand, Ser59 is located at the hydrophilic side of h2 and is a component of the surface Patch II; a hydrogen bond was formed between its hydroxyl group and G1541 of 18S rRNA, and the substitution with Phe would lead to the loss of hydrogen donor (Figure 6C). The detailed structural bases of all the DBA mutations are provided in Supplementary Table 5. In summary, 15.7% (8/51) of DBA mutations may affect structure stability and RPS19-rRNA interactions at the same time. More specifically, 56.7% (17/30) of destabilizing mutations and 47.6% of interaction-disrupting mutations (10/21) may manifest their deleterious impact through more than one approach (Figures 7A,B and Supplementary Table 5).

### Predicting Pathogenicity of New RPS19 Mutations

**FIGURE 8:**
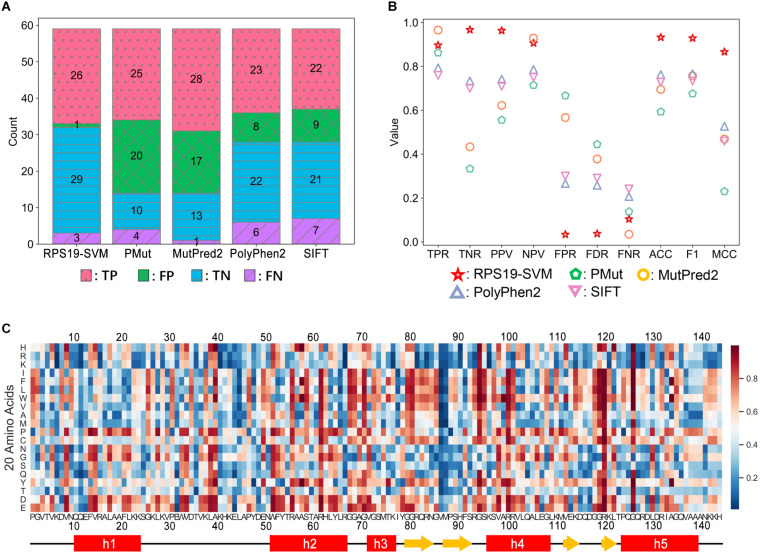
Performance comparison between RPS19-SVM (cross-validation) and other four well-known prediction tools **(A,B)** and the heatmap of RPS19-SVM pathogenicity predictions of all possible mutations of RPS19 **(C)**. The definitions of the performance metrics are described in Supplementary Table 6. The larger the pathogenicity score (ranging from 0 to 1) given by RPS19-SVM, the higher the possibility of being pathogenic.

Our efforts of understanding the structural basis of DBA mutations of RPS19 can provide clues to predict the pathogenicity of newly identified RPS19 mutations. General prediction tools, such as PMut (Lopez-Ferrando et al., 2017), MutPred2 (Pejaver et al., 2017), PolyPhen2 (Adzhubei et al., 2010), and SIFT (Sim et al., 2012), do not perform well with high false positive rate (FPR) on RPS19 mutations (Figures 8A,B), possibly because they internally have the tendency to overestimate the pathogenicity of mutations, as discussed previously (Andersen et al., 2017; Holland et al., 2017; Peng et al., 2018). Another reason may come from that these general tools do not incorporate specific features about RPS19. If we adopt the RPS19 mutations as a specific dataset and incorporate as features the clues in the understanding of DBA mutations, it should be promising to build a better pathogenicity predictor by using machine learning methods, as demonstrated in developing the IDR-specific disease mutation predictor previously (Zhou et al., 2020).

We adopted the support vector machine (SVM) to build the RPS19-specific prediction tool. After further curation, 29 DBA mutations (positive samples) and 30 neutral ones (negative samples) were selected from the mutation dataset as training data. We then extracted the features based on our understandings of the structural basis of DBA mutations. In total, 18 candidate features were extracted for each mutation concerning the interaction with rRNA, structural stability, conservation, etc. After feature selection, 8 features were finally selected (BSA, rBSA, HB_Num, Δcharge, ΔΔG, Δhelix, BLOSUM62, and Δdisorder) (Supplementary Table 7).

Based on fivefold cross-validation, we identified the best hyper-parameters (C = 100, γ = 0.01), and 26 of the 29 DBA mutations can be correctly predicted in the cross-validation, similar to other well-known tools (Figure 8A). At the same time, the FPR has been significantly decreased compared with others (Figure 8A). Overall, much better performance has been achieved as measured by ACC, F1 score, and MCC (Figure 8B). We manually inspected the nine false positives of SIFT (Pro2Leu, Val4Phe, Thr5Pro, Lys24Asn, Asp35Gly, Thr60Ala, Tyr79Cys, Gln105Arg, His145Tyr), and found most of them are located at conserved sites according to the Consurf scores (Supplementary Table 3). According to its prediction logic, SIFT will tend to predict these variants as deleterious. As for the cross-validation of SVM training here, several features independent of conservation have been adopted, which may be responsible for its lower FPR. For these variants, most of them have small or even negative ΔΔG values (Supplementary Table 3), indicating only trivial perturbation of structural stability, which may serve as the reason why the SVM classifier predicts them as neutral.

Finally, we re-trained the predictor, namely RPS19-SVM, on all the training data with the best hyper-parameters, and utilized it to predict the pathogenicity for all possible missense mutations of RPS19 (Figure 8C and Supplementary Table 8). The resulted pathogenicity scores can be valuable in inferring the disease-association of newly identified RPS19 mutations, or the deleteriousness of newly designed mutagenesis mutations.

As ExAC has been upgraded to gnomAD later (Karczewski et al., 2020), we retrieved 25 additional neutral missense variants of RPS19 from gnomAD v2. They were not utilized in the training of RPS19-SVM, so we can use them as an independent testing dataset. It turns out that RPS19-SVM can accurately predict 23 of them as “neutral” with a low FPR of 8% (2/25), much better than the other four tools (PMut: 60%, MutPred2: 76%, PolyPhen2: 20%, SIFT: 40%), confirming its superior performance (Supplementary Table 9). When new positive data are available in the future, further validation could be conducted as well.

Moreover, the heatmap based on the pathogenicity scores (Figure 8C) also confirms our understanding of their molecular mechanisms and can provide new insights. First, mutations substituted by positive residues (Arg, Lys, and His) or by negative residues (Asp and Glu) have relatively low or high pathogenicity scores, respectively. Second, mutations substituted by Proline are not tolerated by RPS19, especially in helix regions. These results are consistent with its characteristics of interaction with negatively charged 18S rRNA and hydrophobic core with a bundle of helices. Moreover, we also found that the two β-hairpin regions, overlapping with the second and third putative IDRs, had lower mutation tolerance for substitution of hydrophobic residues, though few disease mutations in these regions were reported previously. They participate in the composition of surface Patch III and IV, and substitutions by hydrophobic residues may thus affect the interactions with 18S rRNA.

## Discussion

In this work, we conducted a systematic study aiming at revealing the structural basis of DBA mutations at RPS19. Our study illustrated that DBA mutations would disrupt the hydrophobic core related to structural stability or perturb the interactions between RPS19 and 18S rRNA through two or three mechanisms, respectively. Based on these, we further trained an RPS19-specific predictor and predicted the pathogenicity of all possible RPS19 mutations. Logically, the RPS19 molecules bearing DBA mutations would thus be subject to faster degradation or incapability of assembling into the ribosome SSU, resulting in insufficient ribosome quantities and finally DBA symptoms.

Compared with a previous study (Gregory et al., 2007), our work is more comprehensive in that a more complete list of DBA mutations were incorporated, and more specific mechanisms were investigated. Moreover, our studies on several mutations were more reliable. For example, Trp52 and Gly120 were considered to be located at the surface of RPS19, and Trp52Arg and Gly120Ser were thus believed to interrupt the interaction with other molecules according to the previous study (Gregory et al., 2007). However, based on the complex structure of human ribosome, we in this work found that Trp52 is actually almost buried (rSASA = 0.067), and does not participate in the interaction. Moreover, there may exist π–π stacking between Trp52 and nearby residues such as Phe53 and Pro47. The mutation Trp52Arg would decrease the hydrophobicity of this site, and thus destabilize the hydrophobic core, as supported by the calculated ΔΔG (4.35 kcal/mol). As for Gly120, it is actually only partially exposed (rSASA = 0.135), and no interaction can be observed. The mutation Gly120Arg introduces a large and hydrophilic residue, which would decrease the stability of RPS19, as indicated by the ΔΔG calculation (6.51 kcal/mol) as well. Hence, our work has improved or even corrected previous understandings of some DBA mutations.

It should be admitted that our analyses have some shortcomings yet. First, ribosomopathies, including DBA, often manifest in a tissue-specific way, with which two hypotheses have been proposed: the specialized ribosome hypothesis and the ribosome concentration hypothesis (Farley-Barnes et al., 2019). Our work was mainly conducted by following the latter. If the specialized ribosome hypothesis is proved also a major reason for DBA in the future, this kind of systematic analyses should be further improved from this perspective.

Second, in addition to being the indispensable component of ribosome SSU, RPS19 also plays essential roles in many other pathways, including pre-rRNA processing, exportation of SSU precursors from nucleus to cytoplasm, and conformation maturation of 18S rRNA (Leger-Silvestre et al., 2005; Flygare et al., 2007; Juli et al., 2016; Duss et al., 2019). Our work currently only considers the RPS19 mutations’ effects on the maintenance of mature ribosome, and analyses of their perturbation on other pathways of RPS19 should also be conducted in the future.

Third, we only analyzed the structural stability and interactions of RPS19, but the mutations may act from other aspects, such as subcellular localization. Ribosome precursors are first formed in the nucleoli (Phipps et al., 2011; Ameismeier et al., 2018) and then mature in the cytoplasm (Rouquette et al., 2005; Ameismeier et al., 2018). According to immunofluorescence experiments and structural analysis of ribosome intermediates, RPS19 participates in ribosome assembly in the nucleoli (Ameismeier et al., 2018; Klinge and Woolford, 2019), so the RPS19 synthesized in the cytoplasm first needs to enter into the nucleus and be localized in the nucleoli. Two nucleolar localization signals (NoSs)—Met1 to Arg16 and Gly120 to Asn142—in RPS19 have been identified previously (Da Costa et al., 2003). Two DBA mutations—Val15Phe and Gly127Gln—located in these two NoSs, have been proved to fail to localize RPS19 to the nucleoli (Da Costa et al., 2003). Moreover, other mutations like Ala57Pro and Ala61Glu, which are not located in the NoSs, also caused mislocalization of RPS19 (Angelini et al., 2007). Incorporating the effects on subcellular localization of DBA mutations in the future analyses would be beneficial for better understanding of the mechanisms of disease mutations.

It should be noted that the approaches we proposed cannot cover all the DBA mutations. Some residue substitutions are highly likely to disrupt the protein structure, such as Ala17Pro, Ala20Pro, Ala57Pro, Ala58Pro, and Leu64Pro, as they have introduced proline into helical regions. But the free energy changes calculated by FoldX did not support this. Considering that previous studies have suggested that FoldX calculation for mutations containing Pro needs to be optimized (Potapov et al., 2009; Yang et al., 2020), the ΔΔG calculated for these mutations may be far from accurate. Hence, we still treat these mutations as possibly belonging to “Break helix (Mutated to Proline)” (Supplementary Table 5). Another example is Lys23Arg, which may result in an additional intra-molecular hydrogen bond to Leu28, a residue in the IDR located between h1 and h2. Thus, the conformation of this IDR could be restrained, and the interaction between RPS19 and 18S rRNA would be influenced. The summary of the structural bases of those mutations that cannot be covered by our proposed approaches in the section of Results is provided in the column of “Notes” of Supplementary Table 5. It will be promising that the more specific and detailed mechanisms of these mutations are studied by using molecular dynamics simulations in the future.

Except for RPS19, DBA may also stem from defects at other RPs. In our perspective, further studies on the DBA mutations in all related RPs in the future would provide a more comprehensive picture of the pathogenesis mechanisms, which may also shed light on the pathogenesis of other ribosomopathies. Some DBA mutations in other RPs may share similar structural basis as those in RPS19. For example, an N2-Q22 deletion variant of RPS24 is known to cause DBA (Choesmel et al., 2008). We checked the structure of the ribosome complex, and found that the deleted fragment interacts with the h21es6a of 18S rRNA. Hence, it can be assumed that its structural basis may be disrupting the interactions with 18S rRNA. Moreover, considering that haploinsufficiency have been found in many DBA-related RPs (Engidaye et al., 2019), destabilizing protein structure may also be a common mechanism in DBA mutations of these RPs. On the other hand, some RPs are located near the active site in the ribosome complex, so DBA mutations in these RPs may have different mechanisms that were not studied in this work. For instance, RPS26 can bind mRNA molecules during the procedure of translation (Hussain et al., 2014), so some DBA mutations in RPS26 may hold the mechanism of perturbing its binding with mRNA molecules, but not the rRNA.

## Materials and Methods

### RPS19 Structure Analyses

The RPS19 structure was extracted from the coordinates of human ribosome SSU complex (PDB ID: 6G5H) (Ameismeier et al., 2018), and was used as initial conformation for performing molecular dynamics (MD) simulation. The simulation was conducted by using the Amber16 package (Case et al., 2016) with the force field of RSFF2C (Kang et al., 2018), and the explicit solvent model of TIP3P (Jorgensen et al., 1983) was adopted with a periodic box whose edges had a minimum distance of 8 Å to any atom originally presented in the solute.

Before production simulation, energy minimization was performed to relax possible atom collisions, and the system was equilibrated for 2 ns with the final temperature reaching 300.0 K. The production simulation was conducted in an NPT ensemble and lasted 1,000 ns. A timestep of 2 fs was used in both equilibration and production simulation.

The conformations were saved in the trajectory file with an interval of 1 ps, and were analyzed using CPPTRAJ (Roe and Cheatham, 2013). Conformation clustering (dbscan) was performed by setting the backbone RMSD cut-off to 2 Å. The representative conformation of the resulted largest cluster was used for the assignment of secondary structure by using DSSP (Kabsch and Sander, 1983). The 8 states of secondary structure definition were then simplified into 3 states: helix (G, H, I), sheet (B, E), and coil (T, S, C). The backbone RMSF (root mean square fluctuation) of RPS19 was calculated by sampling a frame per 1 ps and using the average conformation as reference. The residues in the middle of the protein with RMSF greater than 2 Å were considered as intrinsically disordered.

The interaction interface of RPS19 in the ribosome complex was identified by using PDBePISA (version 1.48^1^; Krissinel and Henrick, 2007).

### Mutation Data Collection

The DBA missense mutations were collected from dbagenes (Boria et al., 2010), ClinVar (Landrum et al., 2016), and manual literature review. The canonical transcript (ENST00000598742 in Ensembl or NM_001022.3 in RefSeq database) (O’Leary et al., 2016; Yates et al., 2020), was adopted as the reference to map all the mutations. During the data review, several mutations, incorrectly recorded in their original sources, were corrected in this work, including c.182C > A (Ala61Glu), c.358G > C (Gly120Arg), and c.380G > A (Gly127Arg).

The missense variants of RPS19 in the ExAC (Karczewski et al., 2017) were also collected. Based on that the prevalence of DBA is 5–7 in one million individuals, and 25% of them are caused by RPS19 mutations, and the inheritance pattern is autosomal dominant, the minor allele frequency (MAF) of pathogenic RPS19 mutations can be estimated as 6.25E-7 to 8.75E-7. The collected ExAC variants here have allele frequency values with at least one order of magnitude higher. Moreover, considering that DBA usually presents within the first year of life (Ulirsch et al., 2019) and that ExAC has attempted to exclude severe pediatric diseases according to its description (Lek et al., 2016), one can assume that most of these ExAC variants would be neutral. After removing those that has been explicitly recorded as DBA mutations (c.68A > G, c.164C > T, c.208G > A, c.301C > T), the remaining ones served as the neutral dataset in this work.

### Conservation and Electrostatic Potential Calculation

The Consurf score of each site of RPS19 was calculated by using Consurf 2016 server (Ashkenazy et al., 2016). The scores were normalized with mean of 0 and standard deviation of 1. The lower the score, the slower the evolution rate, and the higher the conservation level.

The electrostatic potential on the surface of RPS19 was calculated by using the APBS Electrostatics Plugin integrated in PyMOL (version 2.3.0) with default parameters (Baker et al., 2001; Dolinsky et al., 2004; Schrodinger, 2015).

The Consurf scores and electrostatic potential were rendered at the protein’s solvent-excluded molecular surface with different color schemes by using UCSF Chimera (Pettersen et al., 2004).

### Calculation of Folding Free Energy Change and rSASA

The effect of a mutation on protein structural stability was measured by the change of folding free energy (ΔΔG), and was calculated by using the FoldX package (Schymkowitz et al., 2005). In detail, the RPS19 coordinates from Cryo-EM ribosome structure (PDB ID: 6G5H) was first processed iteratively by using RepairPDB command of FoldX (an energy minimization process) until the decrease of calculated folding energy was lower than 1 kcal/mol in at least 5 iterations. Then we used the PositionScan command of FoldX to mutate all sites to all possible residues, and to calculate the ΔΔG values for all the mutations.

The solvent accessible surface area (SASA) of each residue was calculated by DSSP (Kabsch and Sander, 1983). The relative SASA (rSASA) was obtained by dividing SASA with the maximum SASA, which was computed by placing the specific residue between two Gly residues in an extended conformation accordingly (Tien et al., 2013).

### Predictor Training

The neutral mutations collected from ExAC were treated as negative samples. The positive samples were selected from the DBA mutations if they met any one of these criteria: (1) identified in more than one patient, (2) explicitly annotated as “Pathogenic” or “Likely pathogenic” in ClinVar, and (3) experimentally confirmed to affect the physiological function of RPS19 (expression, nucleolar localization, ribosomal abundance, etc.).

Based on our analyses of DBA mutations, 18 features in four categories were extracted for describing each mutation. The details of these features are listed in Supplementary Table 7.

The feature selection and the SVM hyper-parameter searching were conducted by using the scikit-learn (version: 0.21.3) package in Python. In detail, all the possible feature combinations with at least 5 features were enumerated, and the hyper-parameters C and γ were enumerated from 0.001, 0.01, 0.1, 1, and 100. For each combination of features and hyper-parameters, fivefold cross-validation was run on the training dataset containing the positive and negative samples. When the maximum cross-validation MCC was reached, the optimal feature combination and optimal hyper-parameters were obtained accordingly. The resulted optimal hyper-parameters and features were then utilized to train the RPS19-SVM predictor on all the training data. The predicted results of PMut (Lopez-Ferrando et al., 2017), MutPred2 (Pejaver et al., 2017), PolyPhen2 (Adzhubei et al., 2010), and SIFT (Sim et al., 2012) were obtained by submitting the mutations to their web servers with default settings.

## Data Availability Statement

The original contributions presented in the study are included in the article/Supplementary Material, further inquiries can be directed to the corresponding author/s.

## Author Contributions

KA, Z-QY, and Y-DW conceived the study. KA, J-BZ, YX, and Z-QY collected the data and performed the computational analysis. KA and Z-QY initially drafted the manuscript. Z-QY, Y-DW, WH, and TW revised and proved the manuscript. Z-QY and Y-DW supervised the whole work. All authors contributed to the article and approved the submitted version.

## Conflict of Interest

The authors declare that the research was conducted in the absence of any commercial or financial relationships that could be construed as a potential conflict of interest.

## Footnotes

1 https://www.ebi.ac.uk/pdbe/prot_int/pistart.html
