# Supplementary material for: Computational Studies of the Structural Basis of Human RPS19 Mutations Associated With Diamond-Blackfan Anemia
Source: Front Genet. 2021 May 24;12:650897. doi: 10.3389/fgene.2021.650897 (PMC8181406; doi:10.3389/fgene.2021.650897)
Supplement: Supplementary file 1 [file Image_1.PDF]

Supplementary Material

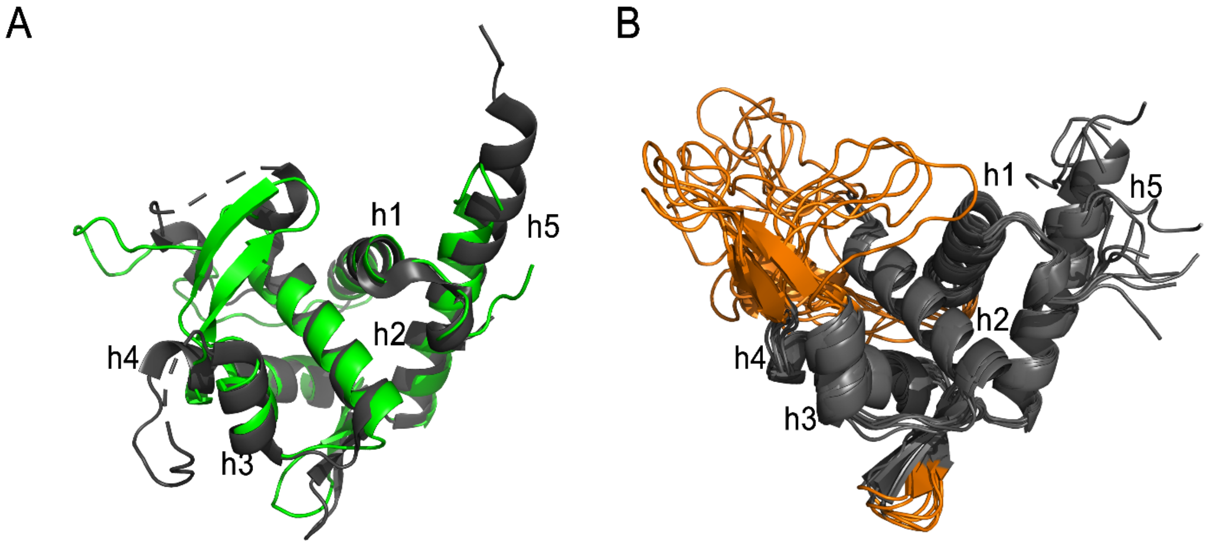

Supplementary Figure 1. The superposition of free RPS19 conformations. **(A)** The superposition of the representative conformation of human RPS19 (green) and the structure of *P. abyssi* RPS19 (grey). **(B)** The superposition of the 7 representative conformations of the conformation clusters. The IDRs were colored in orange. These figures were prepared in PyMOL.
