# Supplementary material for: Computational Studies of the Structural Basis of Human RPS19 Mutations Associated With Diamond-Blackfan Anemia
Source: Front Genet. 2021 May 24;12:650897. doi: 10.3389/fgene.2021.650897 (PMC8181406; doi:10.3389/fgene.2021.650897)
Supplement: Supplementary file 2 [file Image_2.PDF]

frontiers

Supplementary Material

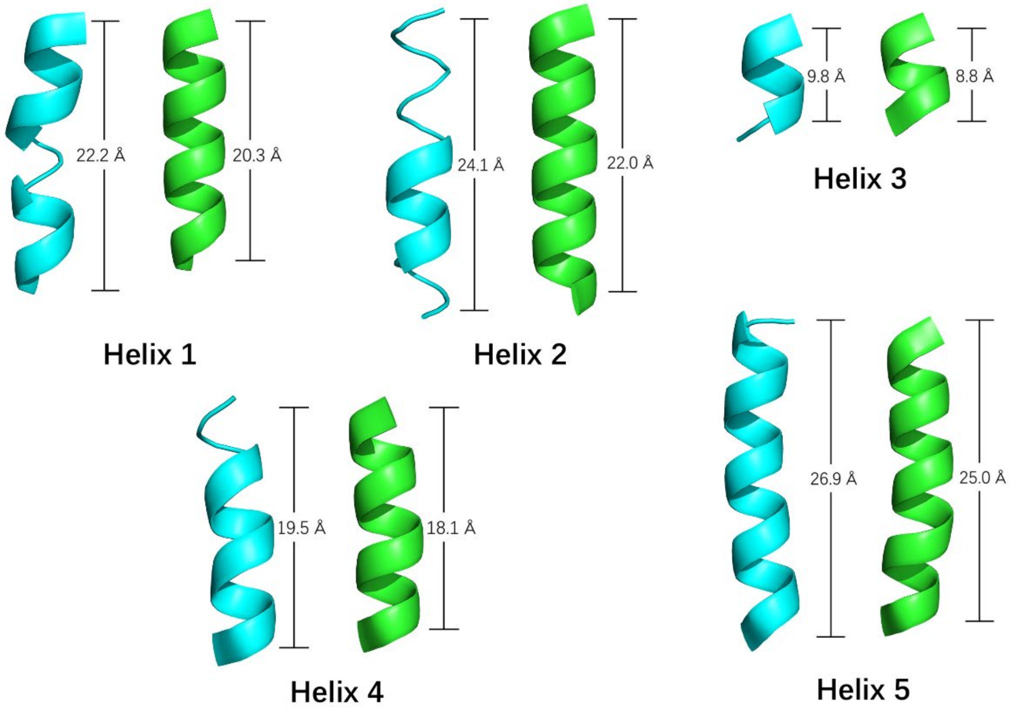

Supplementary Figure 2. The comparison of helix regions from the packed state (blue) and free state (green). The start and end residues of helices were defined according to the free state conformation. The spatial length of helices was measured by the distance between Cα of the two terminal residues.
