# Supplementary material for: Computational Studies of the Structural Basis of Human RPS19 Mutations Associated With Diamond-Blackfan Anemia
Source: Front Genet. 2021 May 24;12:650897. doi: 10.3389/fgene.2021.650897 (PMC8181406; doi:10.3389/fgene.2021.650897)
Supplement: Supplementary file 3 [file Table_1.DOCX]

Supplementary Material

**Supplementary Table 1.** Summary of the Interaction Interfaces of RPS19 in Ribosome SSU Complex

|  | **Interaction partner** | | |
| --- | --- | --- | --- |
|  | **18s rRNA** | **RPS16** | **RPS18** |
| Interface area (Å^2^) | 3029.4 | 287.1 | 467.5 |
| # of RPS19 residues | 75 | 8 | 14 |
| # of partner residues | 61 | 8 | 12 |
| # of hydrogen bonds | 46 | 3 | 5 |
