# Supplementary material for: Computational Studies of the Structural Basis of Human RPS19 Mutations Associated With Diamond-Blackfan Anemia
Source: Front Genet. 2021 May 24;12:650897. doi: 10.3389/fgene.2021.650897 (PMC8181406; doi:10.3389/fgene.2021.650897)
Supplement: Supplementary file 8 [file Table_6.DOCX]

Supplementary Material

**Supplementary Table 6.** Metrics of Performance Evaluation

| **Metrics** | **Description** | **Definition^1^** |
| --- | --- | --- |
| TPR | sensitivity or true positive rate | $TPR=\frac{\mathrm{TP}}{P}=\frac{\mathrm{TP}}{TP+FN}$ |
| TNR | specificity or true negative rate | $TNR=\frac{\mathrm{TN}}{N}=\frac{\mathrm{TN}}{FP+TN}$ |
| PPV | precision or positive predictive value | $PPV=\frac{\mathrm{TP}}{TP+FP}$ |
| NPV | negative predictive value | $NPV=\frac{\mathrm{TN}}{TN+FN}$ |
| FPR | false positive rate | $FPR=\frac{\mathrm{FP}}{N}=\frac{\mathrm{FP}}{FP+TN}$ |
| FDR | false discovery rate | $FDR=\frac{\mathrm{FP}}{FP+TP}=1-PPV$ |
| FNR | miss rate or false negative rate | $FNR=\frac{\mathrm{FN}}{P}=\frac{\mathrm{FN}}{FN+TP}$ |
| ACC | accuracy | $ACC=\frac{TP+TN}{P+N}$ |
| F1 | F1 Score | $F1=\frac{2TP}{2TP+FP+FN}=\frac{2\times PPV\times TPR}{PPV+TPR}$ |
| MCC | Matthews correlation coefficient | $MCC=\frac{TP\times TN-FP\times FN}{\sqrt{(TP+FP)(TP+FN)(TN+FP)(TN+FN)}}$ |

^1^TP: true positive, TN: true negative, FP: false positive, FN: false negative.
