# Supplementary material for: Computational Studies of the Structural Basis of Human RPS19 Mutations Associated With Diamond-Blackfan Anemia
Source: Front Genet. 2021 May 24;12:650897. doi: 10.3389/fgene.2021.650897 (PMC8181406; doi:10.3389/fgene.2021.650897)
Supplement: Supplementary file 9 [file Table_7.DOCX]

Supplementary Material

**Supplementary Table 7.** The 18 Features Used for Feature Selection

| **Category** | **Feature** | **Description** |
| --- | --- | --- |
| Conservation | Consurf_Score | Conservative score of the mutation site, computed by Consurf server (<https://consurf.tau.ac.il/>) |
| Interaction | BSA^1^ | The area that the mutated residue contribute to the RPS19-18S rRNA interface, computed by PDBePISA (<https://www.ebi.ac.uk/pdbe/pisa/>) |
|  | rBSA^1^ | Percentage of BSA compared to the SASA of mutated residue, computed by PDBePISA (<https://www.ebi.ac.uk/pdbe/pisa/>) |
|  | HB_Num^1^ | The number of hydrogen bonds between mutated residue and 18S rRNA, computed by PDBePISA (<https://www.ebi.ac.uk/pdbe/pisa/>) |
|  | WT_Charge, Delta_Charge^1^ | Charge of mutated residue and the difference of charge between before and after substitution |
| Structure Stability | WT_Hydrophobicity,  Delta_Hydrophobicity | Hydrophobicity of the mutated residue, and the difference of hydrophobicity between before and after substitution. The hydrophobicity values comes from ([PMID: 4023714](https://pubmed.ncbi.nlm.nih.gov/4023714/)). |
|  | DDG^1^ | ΔΔG, the difference of folding free energy, computed by FoldX (<http://foldxsuite.crg.eu/>) |
|  | WT_Helix, Delta_Helix^1^ | Helix propensity of mutated residue, and the difference of propensity between before and after mutation. The helix propensity data come from ([PMID: 4358940](https://pubmed.ncbi.nlm.nih.gov/4358940/)). |
|  | WT_Volume, Delta_Volume | Volume of mutated residue, and the difference of volume between before and after mutation. The volume data of residues come from ([PMID: 4566650](https://pubmed.ncbi.nlm.nih.gov/4566650/)). |
|  | Intra_HB_Num | The number of intramolecular hydrogen bonds formed by the mutated residue, computed by hbplus (<https://www.ebi.ac.uk/thornton-srv/software/HBPLUS/>) |
|  | rSASA | Relative Solvent Accessible Surface Area of mutation residue, computed by DSSP (<https://swift.cmbi.umcn.nl/gv/dssp/>) |
| Other | Blosum62^1^ | Blosum62 substitution matrix scores |
|  | WT_Disorder, Delta_Disorder^1^ | Disorder score of the mutated residue, and the difference of disorder score between before and after mutation, computed by Iupred2a (<https://iupred2a.elte.hu/>) |

^1^ features selected for building the final prediction model
